# Supplementary material for: A cost-effectiveness analysis of three surgical options for treating displaced femoral neck fractures in active older patients in Japan: A full economic evaluation
Source: PLoS One. 2024 Oct 29;19(10):e0310974. doi: 10.1371/journal.pone.0310974 (PMC11521282; doi:10.1371/journal.pone.0310974)
Supplement: S1 Table — (DOCX) [file pone.0310974.s001.docx]

**S1 Table. Abridged life table of Japan, 2022 (male).**

| **Age, years** | **Mortality rate** | **Life expectancy** |
| --- | --- | --- |
| 65 | 0.01041 | 19.44 |
| 66 | 0.01147 | 18.64 |
| 67 | 0.01268 | 17.85 |
| 68 | 0.01405 | 17.07 |
| 69 | 0.01565 | 16.31 |
| 70 | 0.01742 | 15.56 |
| 71 | 0.01936 | 14.82 |
| 72 | 0.02146 | 14.11 |
| 73 | 0.02361 | 13.40 |
| 74 | 0.02587 | 12.72 |
| 75 | 0.02843 | 12.04 |
| 76 | 0.03137 | 11.38 |
| 77 | 0.03474 | 10.73 |
| 78 | 0.03863 | 10.10 |
| 79 | 0.04306 | 9.48 |
| 80 | 0.04777 | 8.89 |
| 81 | 0.05320 | 8.31 |
| 82 | 0.05968 | 7.75 |
| 83 | 0.06724 | 7.21 |
| 84 | 0.07592 | 6.69 |
| 85 | 0.08584 | 6.20 |
| 86 | 0.09705 | 5.73 |
| 87 | 0.10946 | 5.29 |
| 88 | 0.12323 | 4.88 |
| 89 | 0.13812 | 4.50 |
