# Supplementary material for: A cost-effectiveness analysis of three surgical options for treating displaced femoral neck fractures in active older patients in Japan: A full economic evaluation
Source: PLoS One. 2024 Oct 29;19(10):e0310974. doi: 10.1371/journal.pone.0310974 (PMC11521282; doi:10.1371/journal.pone.0310974)
Supplement: S2 Table — (DOCX) [file pone.0310974.s002.docx]

**S2 Table. Abridged life table of Japan, 2022 (female).**

| **Age, years** | **Mortality rate** | **Life expectancy** |
| --- | --- | --- |
| 65 | 0.00446 | 24.30 |
| 66 | 0.00480 | 23.41 |
| 67 | 0.00524 | 22.52 |
| 68 | 0.00577 | 21.64 |
| 69 | 0.00636 | 20.76 |
| 70 | 0.00701 | 19.89 |
| 71 | 0.00777 | 19.03 |
| 72 | 0.00871 | 18.17 |
| 73 | 0.00977 | 17.33 |
| 74 | 0.01091 | 16.49 |
| 75 | 0.01219 | 15.67 |
| 76 | 0.01363 | 14.86 |
| 77 | 0.01536 | 14.06 |
| 78 | 0.01747 | 13.27 |
| 79 | 0.02001 | 12.49 |
| 80 | 0.02292 | 11.74 |
| 81 | 0.02635 | 11.00 |
| 82 | 0.03041 | 10.29 |
| 83 | 0.03515 | 9.59 |
| 84 | 0.04061 | 8.92 |
| 85 | 0.04685 | 8.28 |
| 86 | 0.05400 | 7.66 |
| 87 | 0.06237 | 7.07 |
| 88 | 0.07225 | 6.51 |
| 89 | 0.08364 | 5.97 |
