# Supplementary material for: A cost-effectiveness analysis of three surgical options for treating displaced femoral neck fractures in active older patients in Japan: A full economic evaluation
Source: PLoS One. 2024 Oct 29;19(10):e0310974. doi: 10.1371/journal.pone.0310974 (PMC11521282; doi:10.1371/journal.pone.0310974)
Supplement: S3 Table — “Costs after dislocation” in Table 2 is considered as the average costs of “closed reduction for hip dislocation” and “open reduction for hip dislocation”: (42,738+398,620)/2. (DOCX) [file pone.0310974.s003.docx]

**S3 Table. Costs of the currently available surgeries for hip dislocation.**

| **Name of the surgery** | **Healthcare reimbursement code** | **Personnel costs (yen)** | **Price of basic set (yen)** | **Price of special surgical sutures (yen)** | **Personnel costs + non-reimbursable costs (yen)** | **Fee for medical service (yen)** |
| --- | --- | --- | --- | --- | --- | --- |
| Closed reduction for hip dislocation | K061 1 | 22,410 | 20,328 | 0 | 42,738 | 18,000 |
| Open reduction for hip dislocation | K063 1 | 353,740 | 44,880 | 0 | 398,620 | 282,100 |
| Hemiarthroplasty for hip | K081 1 | 265,305 | 44,880 | 23,385 | 333,570 | 195,000 |
| Total hip arthroplasty | K082 1 | 366,940 | 46,380 | 0 | 413,320 | 376,900 |
| Revision total hip arthroplasty | K082-3 1 | 642,145 | 46,380 | 0 | 688,525 | 548,100 |

“Costs after dislocation” in Table 2 is assumed as the average costs of “closed reduction for hip dislocation” and “open reduction for hip dislocation”: (42,738+398,620)/2.
