# Supplementary material for: A cost-effectiveness analysis of three surgical options for treating displaced femoral neck fractures in active older patients in Japan: A full economic evaluation
Source: PLoS One. 2024 Oct 29;19(10):e0310974. doi: 10.1371/journal.pone.0310974 (PMC11521282; doi:10.1371/journal.pone.0310974)
Supplement: S4 Table — IC, incremental cost; ICER, incremental cost-effectiveness ratio; IE, incremental effectiveness; QALY, Quality-adjusted life year. (DOCX) [file pone.0310974.s004.docx]

**S4 Table. Data table of S1 Fig.**

| **Component** | **Quadrant** | **Incremental QALYs** | **Incremental Cost** | **ICER (yen/QALY)** | **Frequency** | **Proportion** |
| --- | --- | --- | --- | --- | --- | --- |
| C1 | IV | IE >0 | IC <0 | Superior | 2021 | 0.2021 |
| C2 | I | IE >0 | IC >0 | ICER <5,000,000 | 4069 | 0.4069 |
| C3 | III | IE <0 | IC <0 | ICER >5,000,000 | 149 | 0.0149 |
| C4 | I | IE >0 | IC >0 | ICER >5,000,000 | 725 | 0.0725 |
| C5 | III | IE <0 | IC <0 | ICER <5,000,000 | 805 | 0.0805 |
| C6 | II | IE <0 | IC >0 | Inferior | 2231 | 0.2231 |

IC, incremental cost; ICER, incremental cost-effectiveness ratio; IE, incremental effectiveness; QALY, Quality-adjusted life year.
