# Supplementary material for: A cost-effectiveness analysis of three surgical options for treating displaced femoral neck fractures in active older patients in Japan: A full economic evaluation
Source: PLoS One. 2024 Oct 29;19(10):e0310974. doi: 10.1371/journal.pone.0310974 (PMC11521282; doi:10.1371/journal.pone.0310974)
Supplement: S5 Table — IC, incremental cost; ICER, incremental cost-effectiveness ratio; IE, incremental effectiveness; QALY, Quality-adjusted life year. (DOCX) [file pone.0310974.s005.docx]

**S5 Table. Data table of S2 Fig.**

| **Component** | **Quadrant** | **Incremental QALYs** | **Incremental Cost** | **ICER (yen/QALY)** | **Frequency** | **Proportion** |
| --- | --- | --- | --- | --- | --- | --- |
| C1 | IV | IE >0 | IC <0 | Superior | 278 | 0.0278 |
| C2 | I | IE >0 | IC >0 | ICER <5,000,000 | 4794 | 0.4794 |
| C3 | III | IE <0 | IC <0 | ICER >5,000,000 | 3 | 0.0003 |
| C4 | I | IE >0 | IC >0 | ICER >5,000,000 | 97 | 0.0097 |
| C5 | III | IE <0 | IC <0 | ICER <5,000,000 | 247 | 0.0247 |
| C6 | II | IE <0 | IC >0 | Inferior | 4581 | 0.4581 |

IC, incremental cost; ICER, incremental cost-effectiveness ratio; IE, incremental effectiveness; QALY, Quality-adjusted life year.
