# Supplementary material for: A cost-effectiveness analysis of three surgical options for treating displaced femoral neck fractures in active older patients in Japan: A full economic evaluation
Source: PLoS One. 2024 Oct 29;19(10):e0310974. doi: 10.1371/journal.pone.0310974 (PMC11521282; doi:10.1371/journal.pone.0310974)
Supplement: S6 Table — The values in pounds sterling (£) and US dollars (US$) were calculated based on the average currency conversion rates in 2022: ¥162 = £1 and ¥131 = US$1. BHA, bipolar hemiarthroplasty; DM-THA, dual-mobility total hip arthroplasty; ICER, incremental cost-effectiveness ratio; SB-THA, single-bearing total hip arthroplasty. (DOCX) [file pone.0310974.s006.docx]

**S6 Table.** **ICER results of scenario analysis A (Japanese yen, pound sterling, and US dollar) stratified by age and sex over a five-year period.**

| **BHA versus SB-THA** | | |
| --- | --- | --- |
| **Age, years** | **Five-year period** | |
|  | **Female** | **Male** |
| 65 | ¥1,518,334 (£9,372; US$11,590) | ¥1,544,124 (£9,532; US$11,787) |
| 70 | ¥1,538,658 (£9,498; US$11,745) | ¥1,561,974 (£9,642; US$11,923) |
| 75 | ¥1,499,440 (£9,256; US$11,446) | ¥1,540,972 (£9,512; US$11,763) |
| 80 | ¥1,465,529 (£9,046; US$11,187) | ¥1,526,412 (£9,422; US$11,652) |
| 85 | ¥1,425,169 (£8,797; US$10,879) | ¥1,534,205 (£9,470; US$11,711) |
| **SB-THA versus DM-THA** | | |
| **Age, years** | **Five-year period** | |
|  | **Female** | **Male** |
| 65 | ¥3,039,119 (£18,760; US$23,199) | ¥3,081,576 (£19,022; US$23,523) |
| 70 | ¥3,006,061 (£18,556; US$22,947) | ¥3,458,102 (£21,346; US$26,398) |
| 75 | ¥4,145,777 (£25,591; US$31,647) | ¥4,529,640 (£27,961; US$34,577) |
| 80 | ¥5,897,579 (£36,405; US$45,020) | ¥6,456,597 (£39,856; US$49,287) |
| 85 | ¥12,170,692 (£75,128; US$92,906) | ¥13,378,666 (£82,584; US$102,127) |

The values in pounds sterling (£) and US dollars (US$) were calculated based on the average currency conversion rate in 2022: ¥162 = £1 and ¥131 = US$1. BHA, bipolar hemiarthroplasty; DM-THA, dual-mobility total hip arthroplasty; ICER, incremental cost-effectiveness ratio; SB-THA, single-bearing total hip arthroplasty.
