# Supplementary figures and images for: A cost-effectiveness analysis of three surgical options for treating displaced femoral neck fractures in active older patients in Japan: A full economic evaluation
Source: PLoS One. 2024 Oct 29;19(10):e0310974. doi: 10.1371/journal.pone.0310974 (PMC11521282; doi:10.1371/journal.pone.0310974)

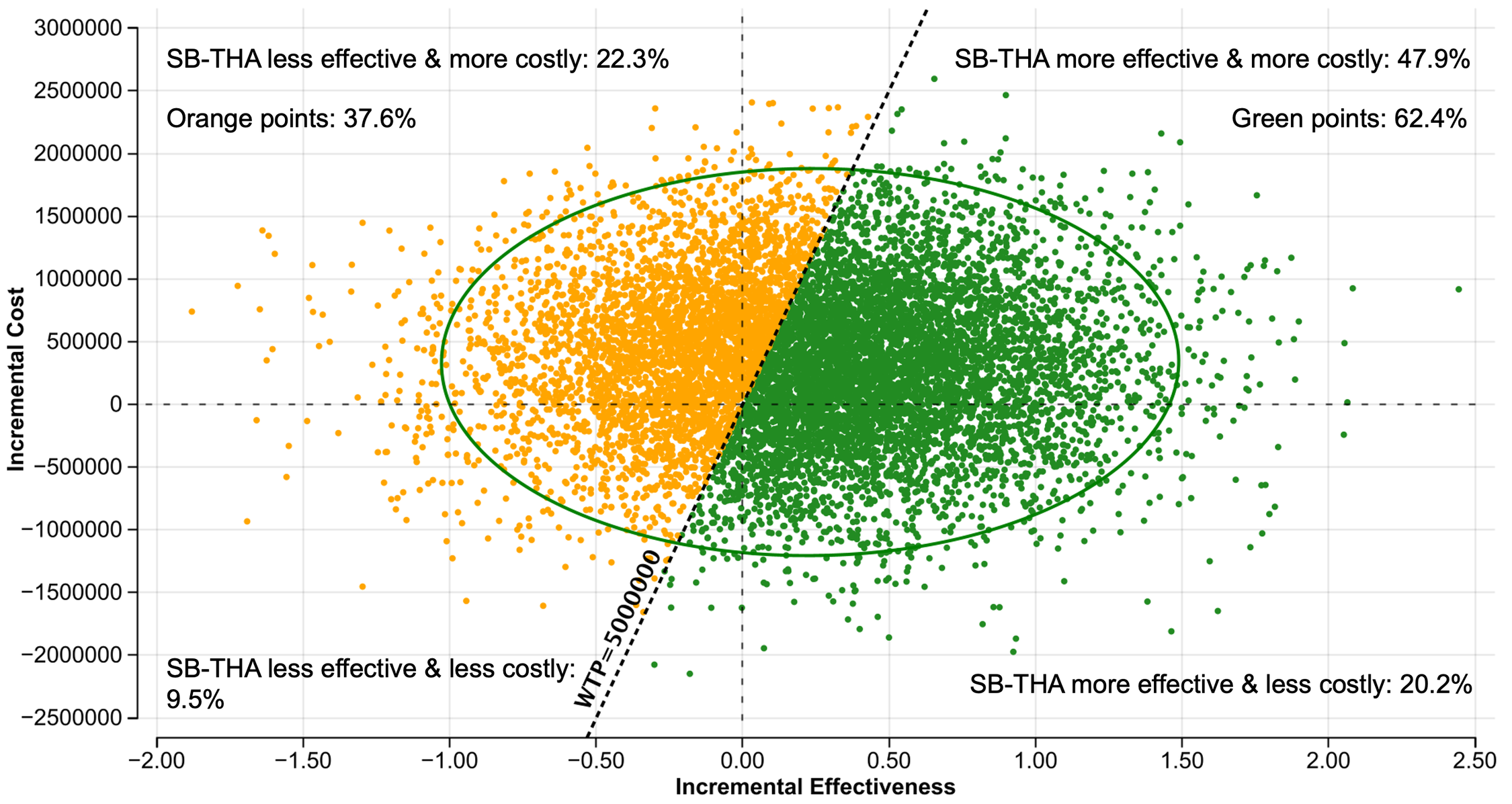

Supplement: S1 Fig — The scatter plot shows outcomes of SB-THA versus BHA in a 75-year-old female patient. Each point represents a single simulated result of 10,000 simulations. The green ellipse denotes the 95% confidence ellipse. BHA, bipolar hemiarthroplasty; SB-THA, single-bearing total hip arthroplasty; WTP, willingness-to-pay. (TIF) [file pone.0310974.s007.tif]

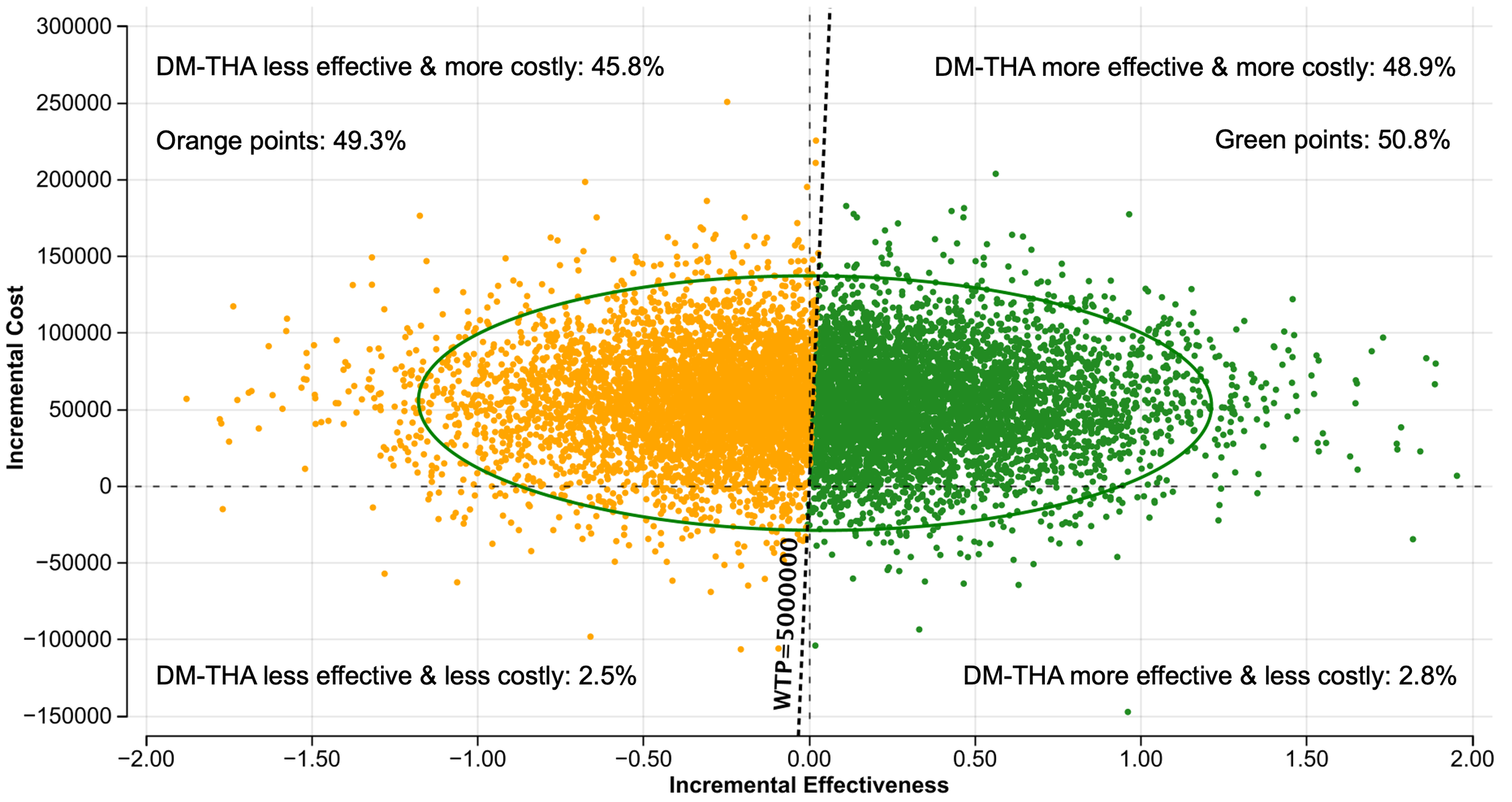

Supplement: S2 Fig — The scatter plot shows cost outcomes of DM-THA versus SB-THA in a 75-year-old female patient. Each point represents a single simulated result of 10,000 simulations. The green ellipse denotes the 95% confidence ellipse. DM-THA, dual-mobility total hip arthroplasty; SB-THA, single-bearing total hip arthroplasty; WTP, willingness-to-pay. (TIF) [file pone.0310974.s008.tif]
