# Supplementary material for: A cost-effectiveness analysis of three surgical options for treating displaced femoral neck fractures in active older patients in Japan: A full economic evaluation
Source: PLoS One. 2024 Oct 29;19(10):e0310974. doi: 10.1371/journal.pone.0310974 (PMC11521282; doi:10.1371/journal.pone.0310974)
Supplement: Attachment — Submitted filename: Response_to_Reviewers.docx [file pone.0310974.s010.docx]

Responses to the Editor’s Comments

Dear Editor:

Thank you for providing us with the opportunity to submit a revised version of our manuscript. We have carefully reviewed the reviewers’ comments and done our best to address their concerns. We hope that you find our work suitable for publication.

Journal Requirements:

When submitting your revision, we need you to address these additional requirements.

**Comment 1:** Please ensure that your manuscript meets PLOS ONE's style requirements, including those for file naming. The PLOS ONE style templates can be found at

<https://journals.plos.org/plosone/s/file?id=wjVg/PLOSOne_formatting_sample_main_body.pdf>

and

<https://journals.plos.org/plosone/s/file?id=ba62/PLOSOne_formatting_sample_title_authors_affiliations.pdf>

**Response:** We have ensured that our manuscript meets PLOS ONE's formatting style requirements. In accordance with the title, author, affiliations formatting guidelines, we have changed the article title (lines 4-6):

*“A cost-effectiveness analysis of three surgical options for treating displaced femoral neck fractures in active older patients in Japan: A full economic evaluation”*

**Comment 2:** Please review your reference list to ensure that it is complete and correct. If you have cited papers that have been retracted, please include the rationale for doing so in the manuscript text, or remove these references and replace them with relevant current references. Any changes to the reference list should be mentioned in the rebuttal letter that accompanies your revised manuscript. If you need to cite a retracted article, indicate the article’s retracted status in the References list and also include a citation and full reference for the retraction notice.

**Response:** We have ensured that the references in our manuscript meets PLOS ONE's formatting requirements. We have updated and modified the references.

Responses to Reviewer 1’s Comments

Reviewer 1

**Comment 1:** Thank you for your work comparing prosthetic options for treatment in femoral neck fractures. The analysis of cost-effectiveness of these options is important in surgical decision making.

**Response:** We appreciate your constructive comments and suggestions. We have revised the manuscript in accordance with your comments. Our responses to your comments have been provided below.

**Comment 2:** Abstract: L25ff: There are more than three surgical options for femoral neck fractures. As you mention below osteosynthesis is a hypothetical option. Hypothetical because in the mentioned age group it is usually not indicated for displaced fractures. Nevertheless, the starting sentence is one of the most important ones, therefore rephrasing is recommended.

**Response:** Thank you for your comments. We agree with your suggestion to rephrase the first sentence of the abstract because the starting sentence is crucial. We have revised the manuscript text as follows (lines 26–34):

*“**For older patients with displaced femoral neck fractures, in which primary osteosynthesis is usually not indicated, there are three primary prosthetic options—bipolar hemiarthroplasty (BHA), single-bearing total hip arthroplasty (SB-THA), and dual-mobility THA (DM-THA). However, the optimal choice for managing displaced femoral neck fractures remains controversial. Accordingly, this study aimed to evaluate the cost-effectiveness of BHA, SB-THA, and DM-THA in active older patients with displaced femoral neck fractures. A decision tree combined with a Markov model was employed to analyze the cost and quality-adjusted life years (QALYs) of BHA, SB-THA, and DM-THA for the management in the Japanese healthcare system.”*

**Comment 3:** Introduction: Good introduction. Register data show much longer follow up periods than the used references. What are the Japanese register numbers?

**Response:** Thank you for the positive feedback. We apologize for the inappropriate explanation, which caused some misunderstanding. We have revised the text in the manuscript as follows (lines 85-86):

*“All data used in this study were collected from books, articles, and open access data from the Japanese Ministry of Health, Labour and Welfare.”*

As stated in this sentence, there are no unique “Japanese register numbers.” This point is considered a limitation of the study. We have added the following sentence in the manuscript (lines 350-351):

*“Registry data regarding these costs are not available in Japan, so further studies using Japanese registry data are needed to address this gap, presenting a challenge for future studies.”*

**Comment 4:** Introduction: L58f: Three primary prosthetic options are generally available. There are a lot more surgical options.

**Response:** Thank you for your comments. We agree with your opinion because there are more than three surgical options, as you mentioned above. We have revised the text in the manuscript as follows (lines 60–63):

*“Considering that primary osteosynthesis is usually not indicated, there are* *three primary prosthetic options generally available for older patients with displaced femoral neck fractures—bipolar hemiarthroplasty (BHA), conventional single-bearing total hip arthroplasty (SB-THA), and dual-mobility THA (DM-THA), which is the most recent surgical option [7,8].”*

**Comment 5:** Methods: L97f: This sentence was already used earlier in the manuscript. Removing here is suggested.

**Response:** Thank you for your correction. We completely agree with your suggestion that a similar sentence is present in the “Ethics statement” (lines 86–87):

*“Because no individual patient data were used, this study did not require patient consent or institutional review board approval.”*

We have removed the sentence: “Institutional review board approval was not required for this study, as it was determined to be a nonhuman study.”

**C****omment 6:** Methods: L117ff: Registry data should be used and could improve the model

**Response:** Thank you for your comment. The Japanese Orthopaedic Association National Registry (JOANR), a database of surgical and therapeutic information for musculoskeletal diseases, was initiated in 2020 but the registry data is currently not accessible for public examination. We expect more complex models to be developed using the registry data in the future, but we believe it unlikely that it would considerably alter the major findings of this study.

**Comment 7:** Methods: L130f: The 5-year mortality rate after hip fracture is 40%. maybe shorter periods should be looked at.

**Response:** Thank you for your constructive comments. As you mentioned, the mortality rate after hip fracture is high. In accordance with your suggestion, we added the results of a scenario analysis. To assess shorter periods, the time horizon was changed from five years to four years, three years, two years, and one year. We have created a new table that describes ICER results (Japanese yen) stratified by time horizon. Please refer to Table 5 (line 287) of the revised manuscript. In addition to Table 5, we added details regarding scenario analyses to the abstract, methods, results, discussion, and conclusion as outlined below.

Abstract (lines 37-38):

*Additionally, two scenario analyses were conducted for other settings.*

Methods (lines 193–197):

*Two scenario analyses (scenario analysis A and scenario analysis B) were performed. First, in addition to the 75-year-old female patient, scenario analyses were conducted for patients of the other age groups and male patients. Specifically, the age range varied from 65 years to 85 years. Second, to assess shorter periods, the time horizon was changed from five years to four years, three years, two years, and one year.*

Results (lines 282–285):

*As in scenario analysis B, the time horizon of the model was changed from five years to four years, three years, two years, and one year (Table 5). SB-THA was more cost-effective than BHA for patients with the time horizon from two to five years. In contrast, DM-THA was more cost-effective than SB-THA for patients only with the five-year time horizon.*

Discussion (lines 313–318):

*In scenario analysis B (Table 5), SB-THA and DM-THA are not cost-effective with a one-year time horizon. This is partly because the cost of implants and the initial year cost are expensive. SB-THA is more cost-effective than BHA with a two- to five-year time horizon thanks to the incremental QALYs. DM-THA is more cost-effective than SB-THA only with a five-year time horizon partly because the relative risk of dislocation of DM-THA compared with SB-THA is estimated to be 0.17 (Table 2).*

Discussion (lines 325-329):

*Third, this study assessed short- and middle-term cost-effectiveness in scenario analysis B. The time horizon was originally set to five years, but we changed the time horizon from five years to four years, three years, two years, and one year. Changing the time horizon broadened the relevance of the study, making it a valuable resource for orthopedic surgeons and healthcare decision-makers.*

Conclusion (lines 370-372):

*For 75-year-old female patients, our results suggest that SB-THA becomes more cost-effective than BHA after two years, and DM-THA becomes more cost-effective than SB-THA after five years.*

We used the latest version of TreeAge Pro Healthcare to conduct additional analyses and have revised the manuscript accordingly (lines 90–93):

*“A decision tree model combined with a Markov model was built using TreeAge Pro Healthcare Version 2024 (Build-Id: 24.2.0-v20240709; TreeAge Software, LLC, Williamstown, MA, United States [US]) to evaluate the cost-effectiveness of three surgical options, conduct one-way sensitivity analyses, and perform probabilistic sensitivity analyses (PSAs) (Fig 1).”*

**Comment 8****:** Results**:** Why did you not present the costs of BHA?

**Response:** Thank you for your question. In addition to Fig 2 and Table 3, we added a few sentences to clarify the costs of BHA in the revised manuscript (lines 205–210):

*“**The cost was* *8,326,071 yen, and the QALYs were 3.792 for the BHA strategy; these figures were* *8,669,497 yen and* *4.021, respectively, for the SB-THA strategy. In other words, SB-THA was more costly than BHA but resulted in more health benefits (measured in QALYs). To explain in more detail, the estimated incremental cost per patient was 343,426 yen, whereas the estimated incremental QALYs were 0.229, resulting in a cost per QALY of 1,499,440 yen/QALY.”*

**Comment 9:** Results**:** L206f: This sentence is discussion. The aware reader should know that already.

**Response:** Thank you for your thoughtful comments. We have deleted the following sentence from the results: “Therefore, both SB-THA and DM-THA are deemed to be cost-effective because they clear the 5,000,000 yen/QALY threshold.” Furthermore, we have modified the first paragraph of the discussion as follows (lines 292–300):

*“**In this study, we conducted a full economic evaluation of three surgical interventions—BHA, SB-THA, and DM-THA—for displaced femoral neck fractures in active older patients in Japan, addressing an important gap in the literature. A* *full economic evaluation is a type of health economic analysis that compares the cost and effectiveness of multiple interventions.* *Compared with BHA, both SB-THA and DM-THA are considered cost-effective for a 75-year-old woman with a displaced femoral neck fracture because they clear the 5,000,000 yen/QALY threshold. The findings of this study suggest that SB-THA could be a cost-effective alternative to BHA and that DM-THA might be more cost-effective than SB-THA for* *relatively younger age groups.”*

**Comment 10:** Results**:** Table 3: Why are data of BHA missing? Deltacost and -QALY is confusing as it is not all the time the compared to BHA.

**Response:** Thank you for your insightful comments. We understand your concern. As you pointed out, the use of delta (Δ) may be confusing for some readers. Thus, we changed “Δ” to “incremental,” and we added “not applicable (NA)” in Table 3. In addition to these changes, we have modified the sentence in the first paragraph of the results (lines 208–210):

*“**To explain in more detail, the estimated incremental cost per patient was* *343,426 yen, whereas the estimated incremental QALYs were* *0.229, resulting in a cost per QALY of 1,499,440 yen/QALY.”*

**Comment 11:** Results**:** L218: I do not understand the following paragraph. Especially the parts about the figure 3. Maybe you could further clarify these figures down to the base for someone who is not very used in these models.

**Response:** Thank you for your thoughtful comments. We understand that some readers may not be familiar with cost-effectiveness analysis and technical terms. Therefore, we have added explanations about technical terms in the manuscript text and Fig 3. Please check the revised paragraph (lines 224–246):

*“Tornado diagrams were generated by changing the parameters of the model base case group. Looking at the ICER for SB-THA compared with that for BHA (Fig 3A), there are* *two* *infinite* *signs and two bars showing threshold values. An infinity sign means that the ICER (“incremental cost”/ “incremental effectiveness”) becomes undefined because the “incremental effectiveness” approaches zero. If “health state utility of well post-BHA” is better than “health state utility of well post-SB-THA,” SB-THA would be “dominated.” If a surgical option is higher in cost but equivalent or lower in effectiveness relative to the comparator, the surgical option is called “dominated.” As for the two bars showing the threshold values, specific values of upper ICER and lower ICER are described. If the initial year cost of SB-THA increases to 2,983,027 yen**, with the other parameters remaining the same, SB-THA would no longer be cost-effective (exceeding the 5,000,000 yen/QALY WTP threshold). Similarly, if the initial year cost of BHA decreases to 1,320,872 yen, with the other parameters remaining the same, SB-THA would no longer be cost-effective. Next, looking at the ICER for DM-THA compared with that for SB-THA (Fig 3B), there are two infinite signs and four bars showing threshold values. If “health state utility of well post-SB-THA” is better than “health state utility of well post-DM-THA,” DM-THA would be “dominated.” In addition, if parameters such as the “relative risk of revision non-dislocation of DM-THA compared with SB-THA,” “relative risk of dislocation of DM-THA compared with SB-THA,” “disutility due to dislocation,” and “health state utility of well post-R-THA” increases to 0.958, 0.348, -0.081, and 0.83, respectively, DM-THA would no longer be cost-effective (exceeding the 5,000,000 yen/QALY WTP threshold). To explain further, for DM-THA to be cost-effective, the relative risk of dislocation should be less than 0.348, meaning that the number of dislocation events after DM-THA would need to be reduced by at least 65.2% compared with those after SB-THA.”*

**Comment 12:** Discussion: 284ff: Generalized sentence. Not needed.

**Response:** Thank you for your insightful comment. Accordingly, we have removed the sentence: “Further studies with a greater number of patients and longer follow-up periods are needed to examine the full effectiveness of these surgical options.”

**Comment 13:** Discussion: L287ff: Because the parameters and the costs of the model are specific for Japan, there is no possibility to translate the results to other countries in any way. Nevertheless, translation of the costs into pounds and dollars is interesting for the international reader. Line 292 until the end of the paragraph is not supported by the data provided!

**Response:** Thank you for your insightful comment. We agree with your comment and have revised the sentences (lines 310–313):

*“**Since the model’s costs are specific to Japan, generalizing the results to other countries is not feasible, and the cost-effective boundary for age might differ depending on the ICER threshold and currency conversion rate in each country.”*

**Comment 14:** Discussion: 302ff: I disagree. The basic data are from Japan. Generalizability to other countries is therefore not given! See also comment above.

**Response:** Thank you for your comment. We have removed the sentence: “Third, converting the results into different currencies broadens the relevance of the study beyond Japan, making it a valuable resource for global healthcare decision makers (S6 Table).”

**Comment 15:** Discussion: 307: The study has some limitations not had.

**Response:** Thank you for the suggestion. We have corrected the word (line 332): “This study also has some limitations.”

**Comment 16:** Discussion: 309f: Long-term studies are always beneficial. Maybe you could argument here with mortality rate as well.

**Response:** Thank you for the constructive comment. Accordingly, we have modified the first limitation (lines 332-339):

*“**First, the longest time horizon of this study was limited to five years, which may not fully capture the long-term effectiveness and cost associated with these surgical options. Long-term follow-up studies would be beneficial for understanding the sustained effects of these interventions. However, if a surgical option is cost-effective over a five-year period, it is likely to remain cost-effective or even* *cost-saving (a dominant strategy) over a longer time horizon, as suggested by a previous study [14]. Additionally, given the five-year mortality rate after a hip fracture in Japan is reported to be 45.6% [32], a five-year time horizon might be considered acceptable.”*

**Comment 17:** Discussion: Additional limitation: The study is not taking into account the state of the patient and decision making in the case of femoral neck fracture. But it is increasing knowledge about cost-effectiveness and therefore further helps the decision process for the patient and his doctor in case of femoral neck fracture.

**Response:** Thank you for the constructive suggestions. We have added the sixth limitation (lines 357–360):

*“Sixth, the study does not consider the patient’s condition or decision-making process involved in treating a displaced femoral neck fracture. However, it does contribute to our understanding of cost-effectiveness, which can be useful in guiding the decisions made by both the patient and the doctor.”*

**Comment 18:** Conclusion: The conclusion about DM THA is not as strong as the advantage of SB THA over BHA. This is not represented in the conclusion sentences. Rephrasing is suggested. BHA is not only an alternative if there are contraindications for THA. Expected mortality of the patient is a very important factor and would change your results.

**Response:** Thank you for the thoughtful suggestion. In accordance with your suggestion (comment 7), we performed scenario analysis B to assess shorter periods. We have rephrased the paragraph in the conclusion section as follows (lines 368–374):

*“**The findings of this study suggest that SB-THA is a more cost-effective option than BHA for patients aged 65–85 years, while DM-THA is a more cost-effective option than SB-THA for patients aged 65–75 years over a five-year period. For 75-year-old female patients, our results suggest that SB-THA becomes more cost-effective than BHA after two years, and DM-THA becomes more cost-effective than SB-THA after five years. Orthopedic surgeons should select the most appropriate surgical option by considering the patient’s expected mortality and overall health condition.”*

**Comment 19:** Conclusion: 339ff: These statements are not supported by the data provided.

**Response:** Thank you for your comment. We have removed the sentence: “Specifically, DM-THA is indicated for relatively younger patients, SB-THA can be considered for active patients aged over 80 years, and BHA is an alternative for patients who have contraindications for THA.”

**Comment 20:** Figure 2: Why is there a line between the points? The datapoints are not fluent. The y axis has to start at 0 so nobody is overestimating the differences.

**Response:** Thank you for the insightful comment and valuable suggestions. We agree that the line between the points is not necessary; therefore, we have removed the lines. Additionally, we have modified Fig 2 in which the Y-axis starts at 0.

**Comment 21:** Figure 3 :I do not understand these graphics. Please further clarify or reduce to important values. Dislocation risk of DM-THA is lower than BHA?

**Response:** Thank you for your comment. We could not reduce the variables because two tornado diagrams were generated using the same variables. To improve clarity in Fig 3, we added the WTP threshold (the yellow line), upper ICER, and lower ICER values, and included a detailed explanation in the figure legend. As indicated by a systematic review [21], the dislocation risk with DM-THA is lower than that with BHA (Relative risk: 0.41).

**Comment 22:** Figure 4: Calculation of willingness to pay is not mentioned in the methods. Could you add this? Why is the willingness to pay of BHA decreasing? This needs discussion. Especially in accordance to mortality rate of old patients and femoral neck fractures.

**Response:** Thank you for your comment. We apologize for the insufficient explanation, which caused some misunderstanding. A willingness-to-pay threshold is a value that estimates how much a patient is willing to pay for a health benefit. It is commonly used in the field of cost-effective analysis. We have added an explanation in the methods section as follows (lines 143–147):

*“The results of the ICER were compared with the established willingness-to-pay (WTP) threshold for cost-effectiveness. A WTP threshold is a value that estimates how much a patient is willing to pay for a health benefit. In general, the Japanese Ministry of Health, Labour and Welfare considers interventions with an estimated ICER of <5,000,000 yen/QALY to be cost-effective.”*

**Comment 23:** Figure S1 and S2: All shortenings need to be clarified in the figure description.

**Response:** Thank you for your suggestion. We have defined the abbreviation “WTP” in the figure legend as “willingness-to-pay.” Additionally, we added an explanation about the green ellipse as follows: “The green ellipse denotes the 95% confidence ellipse.”

Responses to Reviewer 2’s Comments

Reviewer 2

**Comment 1:** Thank you for the opportunity to review this study. This study conducted a full economic evaluation of bipolar Hemi, single bearing THA, or double mobility THA as treatment options for displaced femoral neck fractures in active older patients in Japan. The authors were able to complete an exhaustive cost effectiveness analysis based on an array of costs, assumptions and postoperative risks, which were drawn from Japanese health data and high quality, published clinical research data. While, as an orthopedic surgeon, I have limited ability to assess the correctness of the economic analysis that was performed, I believe the assumptions that were used are reasonable. The cost effectiveness conclusions make sense and our in line with previous literature and our own experiences in Switzerland. This study provides a valuable and high-quality model that quantitatively helps prove the effectiveness of THA vs BHA in displaced femoral neck fractures. It also supports the use of DM in most patients as it likely provides some additional benefit at very limited additional absolute cost, while acknowledging that some uncertainty remains in this regard, which is also in line with the literature.

**Response:** Thank you for your positive evaluation of our study. Two health economists (co-authors) evaluated the economic analysis of this study. We also discussed with a Japanese orthopedic surgeon experienced in treating femoral neck fractures about the assumptions that were used. We are pleased to hear that the assumptions used in our study were considered reasonable by an orthopedic surgeon in Switzerland. A model is a communication tool to reduce the complexity of a particular treatment to its essential elements and to support scientific and rational decision-making. While no model is perfect, we believe our model provides valuable insights and is particularly useful for orthopedic surgeons and healthcare decision-makers.

**Comment 2:** My one recommendation would be to shorten the paragraph between lines 287 and 295. It is not particularly difficult for any reader to convert currency to their own local currency and it should be clear to the reader that depending on current currency fluctuations the precise cost effectiveness threshold may at some points be violated slightly.

**Response:** We really appreciate your valuable comment and agree with your suggestion. Therefore, we have shortened the paragraph as follows (lines 305–312):

*“**The thresholds for cost-effectiveness are set for each country: 5,000,000 yen per QALY for Japan, 50,000 US dollars (US$) per QALY for the US, and 20,000–30,000 pounds sterling (£) per QALY for the United Kingdom [30]. The ICER results of scenario analysis A stratified by age and sex over a five-year period were converted to £ and US$ for reference (Table 4 and S6 Table) using the average currency conversion rate of the 2022 fiscal year (¥162 is equivalent to £1 and ¥131 is equivalent to US$1) [31].* *Since the model’s costs are specific to Japan, generalizing the results to other countries is not feasible, and the cost-effective boundary for age might differ depending on the ICER threshold and currency conversion rate in each country.”*

**Comment 3:** Further, the limitations of the model are well described and honest. I do not, however, believe that they significantly detract from the validity of the findings in this case. As the authors suggest, more complex models could be developed, but they are unlikely to change or adversely affect the major finding of this study.
Finally, the paper is well-written and requires no revision in this regard.

**Response:** Thank you for your valuable comment. As you mentioned, this study has certain limitations. However, to the best of our knowledge, this is the first study to examine the cost-effectiveness of three surgical options for treating displaced femoral neck fractures. We have carefully revised the manuscript and made necessary corrections. We hope that this study offers valuable insights to readers and contributes meaningfully to the field.

**Comment 4:** Overall, this is a well performed study that provides valuable new insights. I thank the authors for their efforts and recommend that the study be accepted for publication with minor revisions. I would perhaps strengthen the conclusions slightly, by making an additional statement that in effect this study supports the use of THA over BHA in all healthy patients over at least a five year period from a cost-effectiveness perspective. However, overall, I would note that the treatment option most appropriate to each patient individually should be chosen.

**Response:** We sincerely appreciate your thoughtful review and positive feedback on our study. Thank you for recommending it for publication with minor revisions. In accordance with your suggestion, we have modified the conclusion as follows (lines 368–374):

*“The findings of this study suggest that SB-THA is a more cost-effective option than BHA for patients aged 65–85 years, while DM-THA is a more cost-effective option than SB-THA for patients aged 65–75 years over a five-year period. For 75-year-old female patients, our results suggest that SB-THA becomes more cost-effective than BHA after two years, and DM-THA becomes more cost-effective than SB-THA after five years. Orthopedic surgeons should select the most appropriate surgical option by considering the patient’s expected mortality and overall health condition.”*

Ultimately, we would like to express our sincere gratitude to the editors and reviewers for their positive and constructive criticism. The manuscript has vastly benefited from your valuable and insightful comments and suggestions. We look forward to hearing from you and would be happy to address any further concerns, if required. We hope this further pushes the manuscript closer to publication in your esteemed journal.
